# Dispositional mindfulness: Is it related to knee osteoarthritis population’s common health problems?

**DOI:** 10.1371/journal.pone.0299879

**Published:** 2024-04-10

**Authors:** Su-Feng Chu, Li-Chen Lin, Aih-Fung Chiu, Hsiu-Hung Wang

**Affiliations:** 1 College of Nursing, Meiho University, Pingtung, Taiwan; 2 College of Nursing, Kaohsiung Medical University, Kaohsiung, Taiwan; 3 School of Nursing, University of Texas, Austin, Texas, United States of America; Iran University of Medical Sciences, ISLAMIC REPUBLIC OF IRAN

## Abstract

**Background:**

A growing body of research supports dispositional mindfulness as important in influencing physical and mental health as well as physical activities in patients with chronic illnesses. Knee osteoarthritis (OA), which often causes health problems, is one of the most common chronic illnesses, but less is known about dispositional mindfulness in relation to this condition.

**Objective:**

To explore possible associations between dispositional mindfulness and physical and mental health as well as physical activity in knee OA patients.

**Methods:**

For this cross-sectional study, we recruited a purposive sample of orthopedic clinic patients in hospitals in Southern Taiwan. Instruments included the Mindful Attention Awareness Scale (MAAS) and the Western Ontario and McMaster Universities Osteoarthritis Index (WOMAC). Health-related characteristics were also measured. Demographic statistics, bivariate correlations, and multiple linear regression informed our exploration of potentially related factors for dispositional mindfulness.

**Results:**

Participants with knee OA (*N* = 250) were mostly elderly (88%), female (70.5%), and married (84%). Marital status, perceived health status, depression, and physical activity were associated with dispositional mindfulness. Better perceived health, lower depression, and greater physical activity were significantly associated with greater dispositional mindfulness. However, the severity of symptoms, fear of falling, and exercise self-efficacy did not reach statistical significance in relation to dispositional mindfulness.

**Conclusion:**

Greater emphasis should be placed on the cultivation of mindfulness to enhance individuals’ perceived health, decrease their depressive mood, and promote their engagement in physical activity, which could indirectly alleviate their experience of pain and improve their physical function, yielding better quality of life and well-being. Future research should focus on interventions to apply dispositional mindfulness in order to determine whether dispositional mindfulness can effectively improve physical and mental health as well as physical activity in those with knee OA.

## Introduction

In recent years, the concept of mindfulness, defined in various ways, has become increasingly important in health care [1, 2]. Mindfulness is ‘‘a process of regulating attention to bring a quality of non-elaborative awareness to current experience and a quality of relating to one’s experience within an orientation of curiosity, experiential openness, and acceptance” [2, p. 234], or the ability to be aware of one’s experiences by paying attention to the present moment in a purposeful, receptive, and nonjudgmental way and thus to one’s own physical and mental processes during ordinary, everyday tasks [1, 3]. It can be considered as a state or as a disposition. *State mindfulness* refers to one’s awareness of momentary conditions—to one’s present experience, moment by moment. *Dispositional mindfulness* suggests that mindfulness is a trait that can be modified through repetitive practice as one conceptualizes being mindful. Individuals’ capacities for dispositional mindfulness vary [3]. Dispositional mindfulness is influenced by one’s environment, but other factors for dispositional mindfulness include genetic and developmental differences [4]. Mindfulness theory thus proposes that dispositional mindfulness is a basic human capacity with variable levels and that it can be trained through mindfulness-based interventions [4].

Increasing evidence from complementary health practices indicates that greater dispositional mindfulness not only reduces stress and stress-related medical symptoms but also enhances physical and mental health and improves physical activity in those with chronic illnesses [5–8]. For example, mindfulness interventions may enhance the acceptance of negative or uncomfortable thoughts and sensations, decrease one’s perception of pain severity, and improve health self-management [9]. Mindfulness interventions can also help individuals better identify and disengage from maladaptive patterns of response, and thus prevent descending spirals of negative thinking and mood [10], reduce mental health symptoms, and prevent the relapse of depression [7, 11]. Mindfulness interventions can contribute to positive affect, improve social connectedness, and help individuals develop interpersonal relationships, as well as alter biological pathways that affect health (e.g., neuroendocrine function, the immune system, and the autonomic nervous system), ultimately improving physical health outcomes [12]. Mindfulness interventions can enable individuals to accept negative sensations likely to occur during physical activity (e.g., fatigue, pain), such that they may enjoy their experience of physical activity with enhanced motivation to engage in it [13–15].

The success of these interventions has led to increased theoretical interest in dispositional mindfulness in relation to chronic conditions. Knee OA is one of the most common chronic illnesses that often cause problems for physical activity as well as physical and mental health [16, 17], and this can have a profound effect on an individual’s quality of life as well as healthcare costs [17]. Knee OA often causes joint pain, joint stiffness, impairment of physical function, and fear of falling, health problems that can lead to depression, reduced self-efficacy for exercise, and engagement in fewer activities [16, 17]. Given prior findings for dispositional mindfulness in relation to chronic conditions, dispositional mindfulness may be particularly useful for addressing problems with physical and mental health as well as physical activity in those with knee OA. However, to the best of our knowledge, research on associations between dispositional mindfulness and common health problems in those with knee OA is limited. We need to know more about these relationships.

In clinical settings, a growing body of research supports an individual’s dispositional mindfulness as an important part of influencing physical, psychological health, and activity behavior in chronic illnesses such as diabetes [5] and cardiovascular events [8, 18]. In the knee OA population, intervention studies have similarly shown that mindfulness training is effective in enhancing physical function, relief of pain [19], improving psychological health [20], and improving physical activity [19]. Most of these studies have tested the effect of mindfulness treatments, but they have not directly and fully assessed the relationship between dispositional mindfulness and common health problems in knee OA, especially perceived physical health, fear of falling, and exercise self-efficacy. Indeed, findings are mixed; a recent mindfulness intervention study on knee OA populations has shown that it may not change dispositional mindfulness or improve health [20].

Thus, the objective of this study is to explore associations between dispositional mindfulness and common problems of physical and mental health as well as physical activity in knee OA patients. We hypothesize that better perceived physical health (i.e., less pain, less joint stiffness, better physical function, and better perceived health status), better psychosocial health (i.e., less fear of falling, lower depression), and better physical activity (i.e., greater exercise self-efficacy, greater physical activity) will be positively associated with greater dispositional mindfulness.

## Methods

### Research design and recruitment

This is a cross-sectional study with purposive sampling. G*Power v3.1.9 was applied to determine a sufficient sample size, with an alpha of 0.05, a statistical power of 0.80, a medium effect size of 0.15, and 12 predictors yielding a minimum sample size of 127. A total of 250 adults completed study questionnaires. The participants were patients diagnosed with OA in orthopedic clinics in hospitals in Southern Taiwan who met the following inclusion criteria and were willing to participate in the study: (a) OA diagnosis by a doctor, and (b) willingness to participate after the study was explained. (c) able to understand and read Chinese; (d) age under 85 years old. Patients were excluded if they (a) were institutional residents; (b) had any cognitive or mental problems, or were blind or deaf; (c) were patients with cancer; (d) had physical limitations caused by diseases other than OA; (e) had undergone or accepted knee OA reconstruction surgery, and (f) regularly performed mindfulness exercises. Nurses were trained to conduct one-on-one interviews with the patients, in which the nurses read questions and guided the patients to read the questions on paper to increase their understanding of the questions’ content. The recruitment period for this study was from Oct 25, 2018, to Feb 28, 2019.

### Ethical considerations

This study was conducted in accordance with the Declaration of Helsinki and approved by the Institutional Review Board of Kaohsiung Medical University (reference number: KMUHIRB-(I)-20180289; date of approval: 24 Oct 2018). Written informed consent was obtained in person from all participants before data collection. Trained nurses were available to answer any questions that came up during the data collection.

### Instruments

#### Dispositional mindfulness

The Mindful Attention Awareness Scale (MAAS) [3] is the most common instrument for measuring dispositional mindfulness. We used the revised Chinese version [21]. This scale measures the level of awareness and attention to experiences in the present moment. The MAAS questionnaire comprises 15 items measured on a 6-point Likert scale from 1 (*almost always*) to 6 (*almost never*). Total scores can range from 15 to 90, with higher scores indicating greater awareness and reception of inner experiences, such that individuals are more mindful of their overt behaviors; higher scores indicate higher levels of mindfulness. The scale has shown good internal consistency (Cronbach’s α = 0.82–0.87) and high test–retest reliability (intra-class correlation = 0.81, *p* < 0.0001) [3].

#### Pain, stiffness and physical function

We used the Western Ontario and McMaster Universities Osteoarthritis Index (WOMAC) for OA self-evaluation [22]. This scale is widely used for patients with OA and has been translated into more than 80 languages. We used the WOMAC pain subscale to measure knee pain (5 items; total scores can range from 0 to 50) and the WOMAC stiffness subscale to measure knee stiffness (two items for joint stiffness; total scores can range from 0 to 20). The WOMAC physical function subscale has 17 items that address difficulty in conducting physical activities (total scores can range from 0 to 170). These three major dimensions are measured with 24 questions; higher total scores signified seriousness of the condition. Cronbach’s alpha was 0.97, and construct validity was confirmed with significant correlations between WOMAC items and a visual analog scale for pain and handicaps (*p* < 0.01) [22].

#### Perceived health

Perceived health represents an individual’s self-evaluated health for specific conditions. For this study, we used the Chinese Perceived Health Status Scale, originally developed from the SF-36 Health Status Scale [23] and revised by Huang and Chiou [24] to include three questions. Respondents provide their self-evaluation of health conditions from 1 year ago to the present, with 1 = *extremely good health condition* and 5 = *extremely poor health condition*. For our analysis, the scores were recoded to positive scoring. Total scores could range from 3 to 15; higher scores indicate better self-evaluated health. Cronbach’s alpha was 0.84 [24].

#### Activities: Specific balance confidence (ABC)

The fear of falling variable was measured with the Activities-Specific Balance Confidence (ABC) Scale [25] to evaluate participants’ confidence regarding balance. The scale has been translated into Chinese, with 16 questions to measure confidence levels in executing indoor or outdoor activities such as passing through parking lots, traveling up and down slopes, mopping the floor, walking up and down stairs, and picking up slippers from the floor [26]. Each item is scored for 0 to 100, from *completely unconfident* to *completely confident*; scores are then averaged for a total score. Scores lower than 50 indicate low confidence in balance function; scores from 50 to 80, medium confidence; and scores over 80, high confidence [26]. Reliability and validity of this scale have been tested, with test–retest reliability of 0.92 and convergent validity from 0.49 to 0.87 [27]. In the present study, higher scores were defined as higher confidence in balance functions; or less fear of falling.

#### Depression

The Geriatric Depression Scale (GDS) [28], modified as a short form with 15 yes/no questionnaire items mostly related to symptoms of depression [29], has been widely applied in older populations. Total scores (0–15) can thus range from no depression to severe. On the Chinese version of the GDS-Short Form [30], scores of 0–4 indicate no depression; 5–8, mild depression; 9–11, medium depression; and 12–15, major depression. With Chinese participants, the scale’s sensitivity and specificity were 96.3% and 87.5%, respectively.

#### Exercise self-efficacy

Self-efficacy for exercise is based on subjective judgments regarding whether an individual can successfully engage in physical exercise [31]. For this study, we applied the Self-Efficacy for Exercise Scale [32] to evaluate levels of confidence in exercising under various circumstances unfavorable to exercise, translated as a Chinese version, the SEE-C [31]. The SEE-C comprises nine questions. Item scores range from 0 = *completely unconfident* to 10 = *completely confident*. Total scores can thus range from 0 to 90, with higher scores indicating greater self-efficacy. Cronbach’s alpha of this scale was 0.75. For criterion-related validity, exercise self-efficacy was positively correlated with physical activity level (*r* = 0.46, *p* < 0.0001) [31].

#### Physical activity

The Physical Activity Scale for the Elderly (PASE) [33] has been translated into Chinese as the PASE-C [34]. The tool measures three types of physical activities: leisure time exercise, household activity, and work-related activity. Leisure time exercise includes five questions on the participant’s involvement in daily activities, such as participating in walking or mild, moderate, or strenuous exercise during the past 7 days. Assessment includes the duration of physical activity per day and the frequency of each level of physical activity per week. Household activity comprises six questions on involvement in daily chores, with “yes” or “no” responses. Scores of leisure-time, household, and work-related physical activities were computed respectively and estimated by multiplying the amount of time spent on 1–2 days (*never*, *seldom*), 3–4 days (*sometimes*), and 5–7 days (*often*) in each activity by item weights, following the scoring manual’s instructions [33]. Higher scores indicate greater physical activity. This scale has been used with Chinese populations (*r* = 0.87). The intra-class correlation was 0.85 (95% CI: 0.81, 0.88) [34].

### Statistical analysis

Data were analyzed with SPSS version 23.0. Descriptive statistics included means, standard deviations, and frequencies. Dispositional mindfulness was examined using independent *t*-tests for the variables of sex, marital status, education. Bivariate correlations were calculated for the variables of age, pain, physical function, perceived health status, fear of falling, depression, exercise self-efficacy, physical activity, and mindfulness. Multiple linear regression was used to explore potentially related factors for mindfulness, with significance set at α < 0.05.

## Results

### Participants’ characteristics and study variables

Participants’ mean age was 68.78 years (*SD* = 7.84); most (70.8%) were female, and 84% were married or with a partner. Most (88.4%) reported a high school education or less. Their average BMI was 25.86 kg/m^2^ (*SD* = 4.25); 60% were overweight or obese. The mean for dispositional mindfulness was 70.12 ± 11.12. The values for other variables for participants are shown in Table 1.

**Table 1:** Participants’ characteristics (*N* = 250).

**Demographic characteristics**	
**Gender, *n* (%)**	
Male	73 (29.2%)
Female	177 (70.8%)
**Age, years (*SD*)**	68.78 ± 7.84
**Marital status, *n* (%)**	
Single/widowed/divorced	40 (16%)
Married/partner	210 (84%)
**Education, *n* (%)**	
≤ High school	221 (88.4%)
> High school	29 (11.6%)
**BMI**	25.86 ± 4.25
Underweight/normal, *n* (%)	100 (40.0%)
Overweight, *n* (%)	150 (60.0%)
**Knee OA health problems**	
**Physical health**	
WOMAC pain	14.01 ± 13.9
WOMAC stiffness	4.75 ± 4.86
WOMAC physical function	41.38 ± 36.60
Perceived health	9.12 ± 3.12
**Mental health**	
ABC	70.00 ± 23.44
GDS-SF	2.3 ± 3.32
**Physical activity**	
SEE-C	35.98 ± 26.46
PASE-C	135.42 ± 74.19

Note: BMI = body mass index; WOMAC = Western Ontario and McMaster Universities Osteoarthritis Index; ABC = Activities-specific Balance Confidence scale; GDS-SF = Chinese version of the Geriatric Depression Scale-Short Form; SEE-C = Chinese version of the Self-Efficacy for Exercise scale; PASE-C = Chinese version Physical Activity Scale for the Elderly.

### Categorical variables and dispositional mindfulness

Participants who were married showed greater dispositional mindfulness than did those who were not married (*p* < 0.001). Participants who were overweight/obese showed greater dispositional mindfulness than did those who were underweight/normal (*p* = 0.034). No significant differences were found for dispositional mindfulness between different gender groups or educational levels. The categorical variables for participants are shown in Table 2.

**Table 2:** Comparison for Mindful Attention Awareness Scale (MAAS) in people with knee OA (*N* = 250).

Demographic characteristics	*n* (%)	*M*	*SD*	*p*
**Gender**				0.367
Male	73 (29.2%)	68.86	14.77	
Female	177 (70.8%)	70.64	13.84	
**Marital status**				<0.001
Single/widowed/divorced	40 (16%)	59.90	12.46	
Married/partner	210 (84%)	72.07	13.58	
**Education**				0.463
≤ High school	221 (88.4%)	69.88	13.79	
> High school	29 (11.6%)	71.93	16.53	
**BMI**				0.034
Underweight/normal	100 (40.0%)	67.80	14.50	
Overweight	150 (60.0%)	71.67	11.12	

Note: *M* = mean; *SD* = standard deviation; BMI = body mass index; *t*-tests were used to compare the means of two groups.

### Correlations

Bivariate correlations between variables are shown in Table 3. Pearson correlations showed that stiffness, perceived health status, exercise self-efficacy, and physical activity were positively correlated with dispositional mindfulness. Depression was negatively correlated with dispositional mindfulness.

**Table 3:** Correlations among continuous variables (*N* = 250).

**Variables**	1	2	3	4	5	6	7	8	9	10
1. Age	1									
2. WOMAC pain	0.05	1								
3. WOMAC stiffness	0.72	0.53**	1							
4. WOMAC physical function	0.10	0.72**	0.70**	1						
5. Perceived health	0.08	-0.18**	-0.21**	-0.25**	1					
6. Fear of falling	-0.12	-0.55**	-0.43**	-0.70**	0.29**	1				
7. Depression	-0.04	0.13*	0.21**	0.24**	-0.37**	-0.29**	1			
8. Exercise self-efficacy	0.01	-0.16*	-0.08	-0.25**	0.37**	0.29**	-0.26**	1		
9. Physical activity	-0.20**	-0.11	-0.02	-0.11	0.22**	0.14*	-0.27**	0.25**	1	
10. Mindfulness	0.06	-0.03	-0.18*	-0.06	0.4**	0.12	-0.44**	0.16*	0.25**	1

Note: WOMAC = Western Ontario and McMaster Universities Osteoarthritis.

*p < 0.05;

**p < 0.01.

### Factors affecting dispositional mindfulness

**Fig 1:**
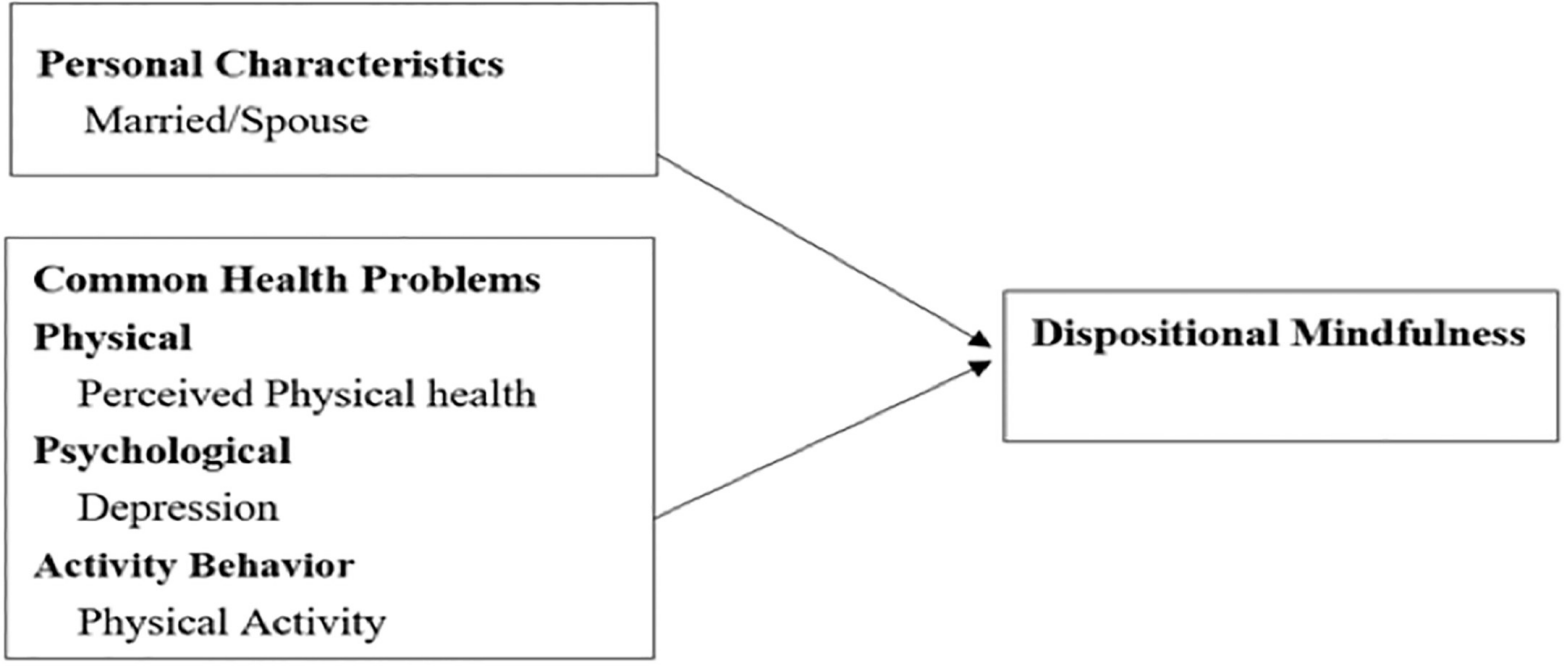
Factors related to dispositional mindfulness.

After adjusting for age, gender, and all significant variables, multiple linear regressions revealed that married/partner, better perceived health, lower depression, and greater physical activity were significantly associated with greater dispositional mindfulness (see Table 4). All variance inflation factors were acceptable, ranging from 1.023 to 1.298. Married/partner participants were significantly related to dispositional mindfulness (β = 8.89, 95% CI [4.827, 12.945], *p* < 0.001). Participants’ perceived health status was significantly associated with dispositional mindfulness (β = 1.26, 95% CI [0.743, 1.790], *p* < 0.001). Depression was significantly negatively associated with dispositional mindfulness (β = -1.20, 95% CI [-1.687, 0.720], *p* < 0.001). Physical activity was related to dispositional mindfulness (β = 0.02, 95% CI [0.000, 0.043], *p* = 0.048). BMI, stiffness, and exercise self-efficacy were without statistical significance in relation to dispositional mindfulness (Table 4). Results for factors related to dispositional mindfulness are also presented in Fig 1.

**Table 4:** Multiple linear regression (adjusting for age and gender) of mindfulness among people with knee OA (*N* = 250).

	B	*SE*	*t*	*p*	95% CI		VIF
					Lower	Upper	
**Intercept**	39.82	4.98	8.00	< 0.001	30.010	49.619	
**Independent variables**							
**Marital status**							
Single/widowed/divorced	ref						
Married	8.89	2.06	4.31	< 0.001	4.827	12.945	1.044
**BMI**							
Underweight/normal	ref						
Overweight	2.16	1.51	1.43	0.154	0.977	1.023	1.023
**Knee OA common health problems**							
**Physical health**							
Perceived health	1.26	0.27	4.65	< 0.001	0.743	1.790	1.298
**Mental health**							
Depression	-1.20	0.25	-4.91	< 0.001	-1.687	-0.720	1.230
**Physical activity**							
Exercise self-efficacy	-0.03	0.03	-0.93	0.352	-0.089	0.032	1.213
Physical activity	0.02	0.01	1.99	0.048	0.000	0.043	1.130

Note: VIF = variance inflation factor. *R*^2 =^ 0.345; adj *R*^2^ = 0.328.

## Discussion

In the knee OA population in this study, greater dispositional mindfulness was significantly associated with being married or with a partner, better perceived health, lower depression, and greater physical activity. However, gender, age, BMI, education, joint pain, joint stiffness, physical function, fear of falling, and exercise self-efficacy were not significantly associated with dispositional mindfulness.

Just as we found that dispositional mindfulness was not associated with gender in those with knee OA, prior studies of cardiovascular health and diabetes have found no significant relationship between dispositional mindfulness and gender [5, 18]. Although we also found that dispositional mindfulness was not related to age in those with knee OA, another study has reported that older adults demonstrate a greater degree of emotional control, are therefore less judgmental, and have a greater awareness of being in the present moment (greater dispositional mindfulness) than do younger adults [35]. This disparate result may be attributable to the fact that knee OA occurs mostly in the elderly; most of our participants were older adults, perhaps resulting in homogeneity. Comparison of dispositional mindfulness between different age groups among those with knee OA is therefore warranted.

Our finding that dispositional mindfulness was not significantly associated with BMI is in line with previous studies of dispositional mindfulness in those with type 2 diabetes [5] and cardiovascular health [18]. In the general health population, greater dispositional mindfulness has been associated with low BMI [36]. Of course, populations with chronic diseases tend to be overweight or obese and show reduced physical activity [37]. Therefore, dispositional mindfulness might not influence BMI in those with a chronic illness such as knee OA. Further studies to compare healthy and knee OA populations are needed.

Our finding that dispositional mindfulness was not associated with education is consistent with previous research on the cardiac disease population [18], and another recent study on the knee OA population found similar results [38]. However, a study with a general population sample differed [39]. This may be due to the focus on a different population (the general population rather than those with knee OA) or to the use of a different tool, the Five Facets of Mindfulness Questionnaire, to evaluate dispositional mindfulness.

Dispositional mindfulness was associated with being married or with a partner in our study. Spouses or partners may provide greater social support as well as romantic relationships, with greater relationship satisfaction leading to greater dispositional mindfulness [40]. This result is incongruent with the result in a study of patients with severe cardiac disease [8], which was a pilot randomized controlled trial with a small sample (*N* = 30). Surprisingly, apart from our result for marital status, we found that dispositional mindfulness in the knee OA population was rarely associated with personal characteristics.

The OA-specific variables of pain, stiffness, and physical function represent the severity of knee OA symptoms. We found no significant relations between dispositional mindfulness and these variables. These findings are dissimilar to those of previous studies with different populations that have shown a relationship between dispositional mindfulness and pain. Most studies agree that greater mindfulness is associated with less interference from pain [6, 19]. One study, for example, has demonstrated that long-term mindfulness-based interventions can significantly increase thresholds for pain and decrease sensitivity to pain [41]. Moreover, many clinicians and researchers consider it essential to target pain in treatment, especially in widely used mindfulness-based intervention therapies for knee OA [6, 19, 42]. Studies strongly confirm the relationship between dispositional mindfulness and pain. Even though one recent study of participants with knee OA reported no direct association between mindfulness and pain, mindfulness did serve as a moderator in that study, suggesting that it may modify how individuals with knee OA cope with pain [20]. Although mindfulness may not change pain, it may impact pain through psychological effects. Thus, mindfulness may not be directly related to pain, but it does impact pain indirectly in knee OA patients. The relationship between dispositional mindfulness and pain needs additional research.

Dispositional mindfulness was not related to OA-specific stiffness, but several mindfulness intervention studies in the knee OA population disagree [38, 42, 43]. Dispositional mindfulness was not related to OA-specific physical function, which is consistent with findings for those with chronic pain (adolescents) [44]. Nevertheless, one study on knee OA patients that disagrees with our results reported that mindfulness-based interventions may improve physical function [43]. A recent systematic review and meta-analysis has also reported that mindful interventions may improve physical function in patients with knee OA [38]. Those studies tested the effect of mindfulness-based interventions on physical function; correlational studies are still needed in order to understand this relationship.

Thus, dispositional mindfulness may be unrelated to knee OA-specific variables for predicting knee OA symptoms’ severity, although mindfulness interventions (such as tai chi and yoga) are widely recommended in treatment guidelines to relieve OA pain and stiffness and to improve physical function and studies do agree [38, 42]. Our results may be attributable to the fact that our participants had lower symptom severity. We recruited them in clinical settings, where they may have received encouragement to engage in physical activity to relieve symptoms. Physical activity would enhance mobility and reduce joint stiffness and pain [45], and healthcare providers may educate these patients on how to manage symptoms and acquire better coping skills [46]. Therefore, symptoms’ severity may not be influenced by dispositional mindfulness in knee OA populations who actively seek professional assistance. Further studies comparing clinical and community knee OA samples are needed.

With regard to the relationship between physical health and dispositional mindfulness, most researchers use objective measurement tools to assess physical health [18, 47], rather than subjective reports of feelings about physical health or perceived physical health. We therefore used a scale for perceived physical health, and our result was similar to the results of previous research in which better perceived health was significantly related to greater mindfulness in diverse samples [6, 48].

As in a previous mindfulness intervention study in older adults [49], fear of falling was not significantly related to dispositional mindfulness in our study. A pilot study did suggest that mindfulness intervention might decrease the fear of falling in knee OA [50], but the study’s small sample may have reflected sampling bias. In another study, fear of falling was reduced after a mindfulness intervention, particularly in older adults with higher fall risk [51]. The participants were not blinded and were informed about all arms of the intervention. Most studies have focused on older adults’ dispositional mindfulness and fear of falling, and not on dispositional mindfulness and fear of falling in the knee OA population. The precise contribution of fear of falling in those with knee OA remains unclear, and further studies are recommended.

Depression is inversely correlated with dispositional mindfulness, and greater dispositional mindfulness is associated with lower depression. According to a literature review, dispositional mindfulness enhances emotion regulation, thus contributing to mental health [52]. A systematic review has also found an inverse association between dispositional mindfulness and depressive symptoms in nonclinical [7] and clinical [11] samples. Recently, a similar result was found with the knee OA population [20]. The results of our study and these studies confirm the inverse relationship between depression and dispositional mindfulness in diverse groups.

Self-efficacy for exercise reflects the individual’s subjective judgments of whether that individual can successfully engage in exercise [32]. We found that dispositional mindfulness was not related to self-efficacy for exercise. Research has reported that dispositional mindfulness in the undergraduate population affects self-efficacy for exercise by increasing intrinsic motivation for exercise through stress management, revitalization, challenges, enjoyment, and health [53]. Also, some studies have reported a positive association between dispositional mindfulness and self-efficacy for exercise in the general population [53, 54]. A recent study on the knee OA population has found that dispositional mindfulness was related to self-efficacy, but it did not focus on self-efficacy for exercise [20]. Research on self-efficacy for exercise in the knee OA population is still lacking.

Our study indicates that dispositional mindfulness is a significant predictor for physical activity, as has been found in studies on cardiovascular disease [18] and knee OA [19]. A systematic review of 20 papers showed a positive relationship between dispositional mindfulness and physical activity [13]. However, the rationale underlying this association remains unknown.

Greater dispositional mindfulness reduces psychological distress, lessens barriers to exercise engagement, and increases exercise motivation [55], which may lead to greater physical activity. In addition, a mindful exercise intervention could not only reduce pain and improve physical function but also enhance physical activity in knee OA [38]. A systematic meta-analysis has reported an effective increase in dispositional mindfulness in older adults with a mean intervention duration of 15.2 weeks and regular practice 2–3 times per week, in various 60-minute mind–body interventions (i.e., Yoga, Tai Chi, Qigong) [56]. In brief, greater dispositional mindfulness is related to greater physical activity, and this relationship may be established when exercise type, duration, and frequency fulfill those criteria. In addition, some studies have demonstrated that dispositional mindfulness may moderate the relationship between intrinsic motivation and physical activity [14, 15]. The relationship between dispositional mindfulness and physical activity is also mediated by psychological factors [13]. Our study provides preliminary evidence that physical activity is positively associated with dispositional mindfulness in knee OA. Further studies are needed to distinguish and explain the role of dispositional mindfulness in relation to physical activity in the knee OA population.

### Implications

Based on the findings of this study, we make recommendations for application in clinical practice. Firstly, healthcare providers can motivate individuals diagnosed with knee osteoarthritis to participate in activities that foster mindfulness, including meditation, yoga, or tai chi. Engaging in these practices has the potential to heighten patients’ sensitivity to their physical well-being, alleviate negative emotions, and elevate their overall physical activity levels. Secondly, given the notable correlation between dispositional mindfulness and marital status or having a partner, healthcare professionals, in the formulation of treatment strategies, should direct attention to the familial and social support systems of patients. Offering positive family support may play a crucial role in nurturing mindfulness among patients and fostering their recovery. In conclusion, this study provides a framework for application in clinical practice to improve the overall health and quality of life for patients with knee osteoarthritis.

## Limitations

The present study has certain limitations. First, it was cross-sectional, and causal inferences or trends cannot be elucidated from the findings. Second, we used only self-report measures, so uncontrolled subjective bias could be present within the findings. Third, survey participants were primarily older adults with knee OA. Their memories might have influenced the study’s results. Fourth, we considered mindfulness as a single dimension, even though mindfulness is multidimensional. Fifth, we recruited knee OA participants who sought treatment from clinics, so their knee OA symptoms might have been relatively mild. Nevertheless, this study’s findings may be important for clinical settings, and they suggest productive directions for further research.

## Conclusion

The data in this study provide key guidance for understanding associations between dispositional mindfulness and health in those with knee OA. Our findings indicate that greater dispositional mindfulness is significantly associated with better perceived health, lower depression, and greater physical activity. Mindfulness should be cultivated to improve perceived health, decrease depressive mood, and engage individuals more in physical activity, which could indirectly reduce their pain, improve their physical function, and provide better quality of life and well-being. We did find no significant correlation between dispositional mindfulness and severity of knee OA symptoms (i.e. physical function, joint stiffness, joint pain), which contradicts findings of previous studies. As a result, we suggest that research should focus on dispositional mindfulness in the knee OA population and compare clinical and community samples. In addition, we found that dispositional mindfulness in the knee OA population is rarely associated with personal characteristics other than marital status. Further research on marital status and dispositional mindfulness is needed. Finally, future research must focus on interventions involving the application of dispositional mindfulness to determine whether dispositional mindfulness effectively improves the physical and mental health as well as physical activity in those with knee OA.

## Supporting information

S1 File (DOCX)

S1 Data (XLSX)
