# Supplementary material for: Dispositional mindfulness: Is it related to knee osteoarthritis population’s common health problems?
Source: PLoS One. 2024 Apr 10;19(4):e0299879. doi: 10.1371/journal.pone.0299879 (PMC11006190; doi:10.1371/journal.pone.0299879)
Supplement: S1 File — (DOCX) [file pone.0299879.s001.docx]

**特質正念：它是否與膝關節炎患者的常見健康問題相關?**

**摘要**

背景：越來越多的研究支持特質正念對於影響患有慢性疾病患者的身體和心理健康以及身體活動的重要性。膝蓋骨關節炎（OA）是最常見的慢性疾病之一，常引起健康問題，但有關這種狀況與特質正念的相關性知之甚少。 目標：探討特質正念與膝蓋骨關節炎患者的身體和心理健康以及身體活動之間的關聯性。 方法：這是橫斷面研究，我們在台灣南部的醫院招募了骨科門診患者樣本。使用的工具包括《正念注意觀察量表》（MAAS）和《西安大略和麥馬士大學骨關節炎指數》（WOMAC）。還測量了健康相關特徵。我們通過人口統計學、雙變量相關和多元線性迴歸來探討可能與特質正念有關的因素。 結果：參與膝蓋骨關節炎的受試者（N = 250）大多是老年人（88％），女性（70.5％），已婚84％）。婚姻狀況、感知健康狀態、抑鬱和身體活動與特質正念相關。更好的感知健康、較低的抑鬱和更大的身體活動與較高的特質正念呈現顯著相關。然而，症狀的嚴重程度、對跌倒的擔憂和運動自我效能感在與特質正念的相關性方面並未達到統計學上的顯著水平。 結論：應更加強調培養正念，以增強個體對健康的感知，減輕抑鬱情緒，並促進身體活動的參與，這可能間接緩解疼痛體驗，改善身體功能，提高生活質量和福祉。未來的研究應該集中於應用特質正念的介入措施，以確定特質正念是否能夠有效改善膝蓋骨關節炎患者的身體和心理健康以及身體活動。

**關鍵詞**: 膝關節炎，特質正念
